# Tunable Plasmonic Bandwidth Broadening via DC Electrical Bias

**DOI:** 10.3390/nano15110794

**Published:** 2025-05-25

**Authors:** Chen Wei, Fuhua Gao, Fan Yang

**Affiliations:** Key Laboratory of High Energy Density Physics and Technology of the Ministry of Education, College of Physics, Sichuan University, Chengdu 610065, China; weichen@stu.scu.edu.cn

**Keywords:** direct current electrical modulation, bandwidth broadening, quantum hydrodynamic theory, surface charging

## Abstract

The ability to broaden the bandwidth of nanodevices holds significant promise for applications in modern science and technology. In this work, we demonstrate a tunable approach to the bandwidth modulation of nanoresonators by applying a direct current electric field. Quantum hydrodynamic theory reveals that the biased electric field redistributes surface charges, inducing positively and negatively charged regions on the metal surface. This charge asymmetry splits the plasmonic modes, resulting in bandwidth broadening. The optical response can be finely tuned by varying the amplitude and polarization direction of the bias field. This mechanism offers a versatile strategy for developing nanodevices, including metasurfaces with dynamically adjustable bandwidths.

## 1. Introduction

Bandwidth is a crucial parameter for assessing the optical performance of devices, garnering extensive interest from researchers across diverse fields. In nanophotonics, strategies for enhancing light–matter interactions often revolve around material selection and structural geometry optimization. On the materials side, bandwidth broadening has been achieved through heavily doped semiconductors, where free carrier density and filling factors are carefully controlled [1]. Similarly, materials like chromium, whose admittance closely matches that of air, have enabled broadband high absorptance in the visible spectrum by serving as admittance-matching layers [2].

However, the inherent limitations of material dispersion restrict the applicability of these approaches, particularly for broadband applications such as perfect absorbers and achromatic lenses. Structural geometry engineering offers greater flexibility, with metamaterials and metasurfaces emerging as excellent platforms for designing broadband optical devices [3,4,5]. For example, broadband responses can be achieved by creating unit cells with multiple resonances, often using supercells composed of varying structures [6]. In imaging applications, broadband achromatic metalenses compensate for dispersive phase differences by tailoring the geometries of individual unit cells [7,8,9].

In plasmonic systems, where light interacts with matter at the nanoscale [10], singular structures—such as sharp tips or narrow metal–insulator–metal gaps—have been shown to broaden bandwidths effectively [11]. These structures, such as two kissing particles [12] or singular metasurfaces [13], mimic continuous spectra from infinite slab geometries, enabling broadband light harvesting. An excellent application of the singular structure is “Black Gold”; the original shining golden surface becomes black when the surface is structured with an array of ultra-sharp convex grooves, making the light substantially absorbed in the whole visible domain [14].

However, the bandwidth of these methods is inherently fixed, as it depends on the structure’s geometry or material composition. One optional approach integrates the magneto-optical response of magnetic materials with the local field enhancement effect of plasma resonance, achieving broad-spectrum tunability through the synergistic control of polarization and particle spacing [15]. However, this method involves indirect tuning. Recent advancements in the dynamic tuning of plasmonic resonances via direct current (DC) electrical modulation offer a promising alternative [16,17,18]. Li et al. demonstrated that electrical bias creates nanoscale electron reservoirs, enabling tunable plasmonic responses [16]. De Luca et al. showed how static bias enhances free-electron third-harmonic generation by modulating surface charges [17]. Zurak et al. studied how direct electrical charging influences the amplitude, resonance frequency, and linewidth experimentally and theoretically [18]. Applying a DC bias directly charges the metal surface through electrostatic screening, shifting resonance peaks depending on the bias polarity. A positive bias results in a blueshift, while a negative bias induces a redshift in the optical response [19,20,21].

In this paper, we propose a novel approach to achieve tunable bandwidth broadening in plasmonic nanoresonators by incorporating a DC bias field. Here, we demonstrate how a DC bias field can polarize nanoresonators to achieve dynamic, tunable bandwidth broadening. Using quantum hydrodynamic theory (QHT), we elucidate the underlying physical mechanism and validate the approach by demonstrating substantial modulation of the optical response. Finally, we show how this mechanism can be applied to create plasmonic metasurfaces with adjustable bandwidths, offering new opportunities for tunable nanophotonic devices.

## 2. Methods and Results

**Figure 1:**
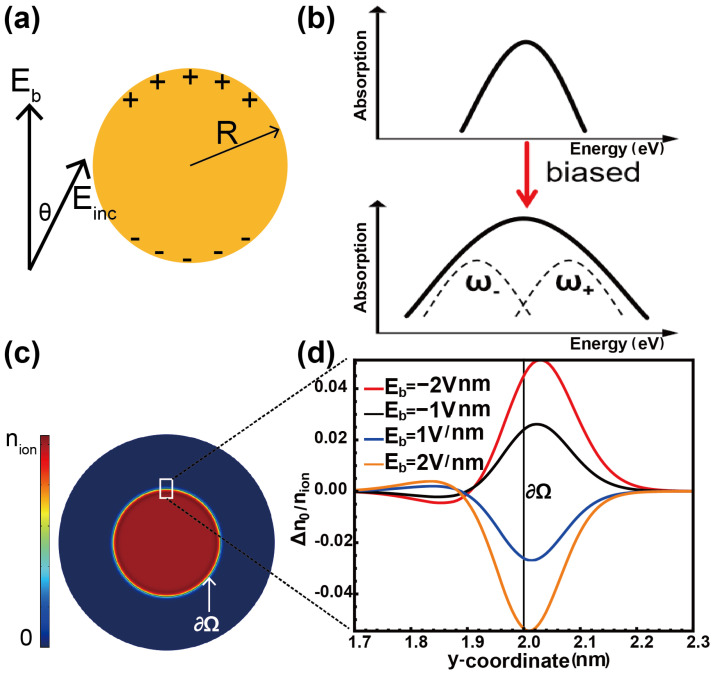
Schematic diagram for applying a biased electric field. (**a**) Schematic of charge distribution after biasing. The upward arrow indicates the direction of the bias electric field, and $E_{inc}$ is the direction of the incident field. The angle between $E_{inc}$ and $E_{b}$ is θ. (**b**) Conceptual illustration of how the bias affects the bandwidth of spectra. The upper column indicates when no bias is applied, while the lower column indicates when bias is applied. (**c**) The electron distribution of the ground state $n_{0}\left(r\right)$. (**d**) Electron density variation $\Delta n_{0}=n_{0}{{|}_{bias}-n_{0}|}_{no\;bias}$ across the top of the metal surface ∂Ω. Throughout this paper, the radius of the nanowire *R* = 2 nm.

To illustrate the mechanism of DC-biased field-induced bandwidth broadening, we consider a prototypical nanowire resonator subjected to a DC electric field $E_{b}$, as depicted in Figure 1. The simple 2D nanowire case is employed as an example that captures the main physics of our ideas while simultaneously reducing a substantial computational cost. Nanoresonators with narrow bandwidths are chosen as the starting point, excluding singular structures that inherently exhibit broad spectra. When the DC field is applied, an electrostatic screening effect generates a screening field within the metallic nanowire, displacing free electrons relative to the positive ion background. This displacement creates non-uniform surface charges: The top surface becomes positively charged, while the bottom becomes negatively charged (Figure 1a). Although the nanowire remains globally charge-neutral, this redistribution of surface charge splits the plasmonic resonances. This differs from previous works, which involve plasmonic nanoresonators that are either positively or negatively charged. Positive charges on the surface blueshift the resonance peak, while negative charges redshift it, resulting in spectral mode splitting and bandwidth broadening (Figure 1b) [19,20].

The electron displacement induced by the DC field occurs on the mesoscopic scale. Given that the system’s radius studied in this paper is just a few nanometers, it becomes necessary to include the quantum effects of electrons [22,23]. In addition, since the induced charge is primarily concentrated at the metal interface and classical bulk model [18] overlooks the quantum behavior of electrons at this boundary, it fails to accurately capture the variations in the optical response. To account for myriads of nonclassical effects of electrons in metallic nanostructures, such as nonlocality and electron spill-out, we employ the QHT approach to capture these nonclassical effects for a sodium nanowire [24,25,26,27].

We selected QHT as our primary theoretical tool because it can accurately reproduce the results of ab initio time-dependent density functional theory (TDDFT). This is attributed to the two theories’ common foundation in the density-functional framework for many-body systems and their use of the same Kohn–Sham (KS) density [26]. Compared to TDDFT, the advantage of QHT lies in its ability to efficiently and intuitively describe the nonlocal effects, collective behavior, and dynamical response of nanostructures, which is particularly applicable to optical problems [24,25,26,27]. Regarding computational cost, QHT scales at $O(N_{e})$, which is significantly more efficient than TDDFT’s $O(N_{e}^{3})$ scaling.

As a simple metal, sodium has been widely used as a prototype to study nonclassical effects for plasmonic systems, whose ion density is $n_{ion}$ = $\frac{3}{4\pi {\left(r_{s}a_{0}\right)}^{3}}$ with $r_{s}$ = 4 ($a_{0}$ is the Bohr radius) [28]. The basic assumption of the hydrodynamic model is that metal can be treated as a many-body electronic system described by the electron density *n* and the electron velocity field v, whose dynamic can be expressed by [26,29]:

(1) $$m_{e}\left(\frac{\partial }{\partial t}+v\cdot \nabla +\gamma \right)v=-e\left(E+v\times B\right)-\nabla \frac{\partial G[n]}{\partial n}$$

where $m_{e}$ and *e* are electron mass and charge (in absolute value), respectively, and γ is the damping rate, here taken as 0.066 eV/ℏ. The energy functional G[n], which plays a pivotal role in QHT, is expressed as $G\left[n\right]=T_{TF}\left[n\right]+\lambda T_{W}\left[n,\nabla n\right]+E_{XC}\left[n\right]$ [26]. Here, $T_{TF}\left[n\right]$ represents the Thomas–Fermi kinetic energy functional, $T_{W}\left[n,\nabla n\right]$ denotes the von Weizsäcker term, and $E_{XC}\left[n\right]$ corresponds to the exchange-correlation energy functional within the local density approximation. The specific expression of G[n] can be found in Ref. [26]. The parameter λ, which weights the von Weizsäcker functional, is critically important as it governs the decay of the electron density. Typically, λ is chosen within the range 1/9 ≤ λ ≤ 1 [29]. In this study, we adpot λ = 1/9.

For the ground state, the equations can be expressed as [24,25]:

(2) $$\begin{array}{c} {\left(\frac{\partial G[n]}{\partial n}\right)}_{0}-e\phi_{0}=0\\ \nabla^{2}\phi_{0}+\frac{e}{\epsilon_{0}}\left(n_{+}-n_{0}\right)=0 \end{array}$$

in which $\phi_{0}$ is the electrostatic potential and $n_{+}$ is the jellium background density that equals ion density $n_{ion}$ inside the jellium edge but vanishes outside. $n_{0}$ is the ground-state electron density to be determined by Equation (2).

For the excited state,

(3) $$\begin{array}{c} \nabla \times \nabla \times E-\frac{\omega^{2}}{c^{2}}E=\omega^{2}\mu_{0}P\\ \frac{en_{0}}{m_{e}}\nabla {\left(\frac{\partial G[n]}{\partial n}\right)}_{1}+\left(\omega^{2}+i\gamma \omega \right)P=-\frac{n_{0}e^{2}}{m_{e}}E \end{array}$$

where the hydrodynamic equation is coupled with Maxwell’s equation [26].

When the biased dc electric field $E_{b}$ is applied to the nanoresonator, the boundary condition $-\nabla \phi_{0}{|}_{\partial \Omega }=E_{b}$ is incorporated into the QHT calculation, where ∂Ω represents the boundary for the calculation domain for electrostatic potential $\phi_{0}$ [16]. By solving the system of equations with these boundary conditions in the finite element solver Comsol Multiphysics [30], both the ground and excited states under the influence of the DC bias can be numerically determined. Additionally, the biased DC electric field can be achieved through a parallel-plate electrode configuration [31].

The application of a DC bias field modulates the ground state electron distribution, which in turn affects the excited state. The electron density profile $n_{0}\left(r\right)$ of the ground state, shown in Figure 1c, gradually decreases from the ion density $n_{ion}$ to zero across the jellium edge ∂Ω of the nanowire. To assess the impact of a biased DC field on the ground state, Figure 1d presents the distribution of the charge density variation ($\Delta n_{0}=n_{0}{{|}_{bias}-n_{0}|}_{no\;bias}$). In cases where only nonlocality or the classical theory is considered, the ground state charge distribution behaves as a step function, uniformly distributed within the metal, and is not adjustable by the biased field. A detailed examination of the metal interface ∂Ω at the nanowire’s top, as shown in Figure 1c, provides insight into the electron density change under different electric field biases. The increase in the bias field leads to enhanced charge accumulation at the boundary, thereby exerting a more pronounced influence on the excited state.

The profile aligns with the conceptual illustration in Figure 1a, showing that the applied biased field charges the metal surface. Under a positive bias, $E_{b}$ is polarized as indicated in Figure 1a, and the electron density decreases ($\Delta n_{0}<0$), rendering the surface positively charged. Conversely, a reversed bias leads to an increase in electron density, resulting in a negatively charged surface. This dependence of electron density variation $\Delta n_{0}$ on the biased field $E_{b}$ demonstrates the tunability of the surface charging effect.

**Figure 2:**
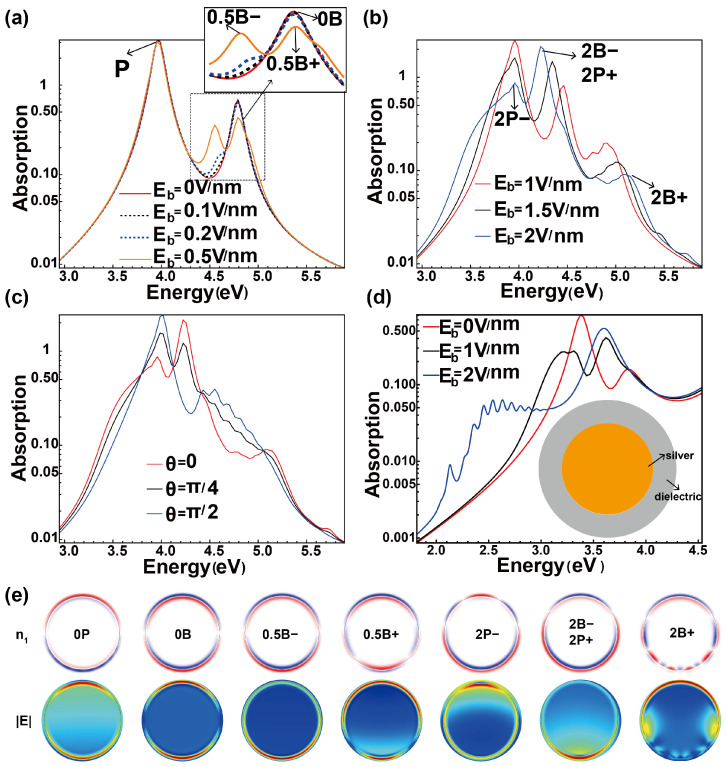
Influence of the biased electric field on the optical response of a single nanowire. Different colors represent different values of the bias electric field. (**a**,**b**) Absorption efficiency [35] spectra corresponding to different values of bias electric field $E_{b}$. The incident and bias electric fields are in the same direction. (**c**) Absorption efficiency spectra correspond to different angles θ between the incident electric field and the bias electric field. The biased electric field is 2 V/nm. (**d**) Absorption spectra of silver nanowire coated by a thin layer of silica corresponding to different values of bias electric field $E_{b}$. The inset shows the schematic of the structure. The coating has a thickness of 2 nm and an permittivity of 2. (**e**) Charge distributions (**top row**) and electric field distributions (**bottom row**) of resonant peaks corresponding to different values of the biased electric field. “P” and “B” represent the plasmon mode and the Bennett mode, respectively. The number in front of “P” and “B” indicates the value of the biased electric field. “B+” and “B−” denote the two peaks splitting of the Bennett mode by the positive and negative charges, respectively.

The modulation of the ground state directly influences the excited state, manifesting as changes in the optical response. Figure 2 illustrates the optical behavior of the nanowire under a DC-biased field. Without the application of a DC electric field, the absorption spectrum of a single nanowire, shown in Figure 2a, exhibits two distinct resonance peaks corresponding to the dipole surface plasmon (SPP) mode and the Bennett mode [32,33]. The Bennett mode, originating from the oscillation of induced electron at the metal surface, has been experimetally observed on smooth films of potassium and sodium [34]. The charge density distributions and electric field distributions for these two modes are displayed in Figure 2e, labeled as “0P” and “0B”, where “0” denotes the absence of bias, and “P” and “B” refer to the SPP and Bennett modes, respectively. The primary difference between these modes lies in the charge distribution at the metal interface. For the SPP mode, the induced charge is predominantly positive or negative on the metal surface. In contrast, the Bennett mode features an equal distribution of positive and negative charges across the metal interfaces, forming a surface dipole layer. The electric field distributions demonstrate that in both modes, field enhancement is localized at the nanowire surface. Particularly, the Bennett mode displays a more confined electric field.

In Figure 2a, as the bias electric field increases to 0.5 V/nm, the Bennett mode broadens and begins to split, while the conventional SPP mode shows no detectable changes. This indicates that the Bennett mode is more sensitive to variations in charge distribution at the metal interface. At a bias field of 0.5 V/nm, the original Bennett mode separates into two distinct peaks, as shown in Figure 2e. These peaks are labeled as “0.5B−” and “0.5B+”, corresponding to the redshifted mode induced by negative charges and the blueshifted mode induced by positive charges, respectively. The associated charge profiles reveal prominent surface dipole layers at the bottom and top metal interfaces, resulting from the opposing surface charges. Similarly, the field distribution changes. The field enhancement of the redshifted mode is particularly localized near the lower surface of the nanowire.

As the electrostatic bias increases further, the SPP mode initially broadens and eventually splits when the bias field reaches 2 V/nm, as shown in Figure 2b. The charge density distributions and field distributions corresponding to the three main spectral peaks at this bias field are illustrated in Figure 2e. Notably, the highest peak, observed near 4.2 eV, is a superposition of the blueshifted SPP mode (“2P+”) at the top interface and the redshifted Bennett mode (“2B−”) at the bottom interface, with the two modes being spatially distinct.

The angular dependence of the absorption spectrum was also investigated. Figure 2c shows how the absorption spectrum changes with the angle θ between the incident field and the biased electric field when $E_{b}=2$ V/nm. When the two fields are perpendicular, the absorption spectrum exhibits a narrower bandwidth and smaller resonance peak shifts compared to when the fields are aligned. At θ=0, the region of electric field enhancement aligns optimally with the area of maximum electron variation, resulting in the greatest bandwidth broadening. Thus, in addition to the biased field amplitude, the polarization direction provides an additional degree of control for tuning the spectrum bandwidth.

In practical plasmonic systems, noble metals like gold and silver are more commonly used; however, they do not exhibit the Bennett resonance [24]. But this can be achieved by using a dielectric coating [29]. Additionally, the coating serves as a protective layer in experiments to prevent oxidation.

Silver, for example, has interband transitions from the filled 4d-band to the 5s-band, so it does not strictly follow the Drude model in the high-frequency region. Take the interband transitions into consideration for silver, and the plasmonic mode can be tuned by applying a bias electric field, as illustrated in Figure 2d, with the inset showing a schematic of the structure. This highlights the universal applicability of DC bias field modulation for bandwidth broadening.

**Figure 3:**
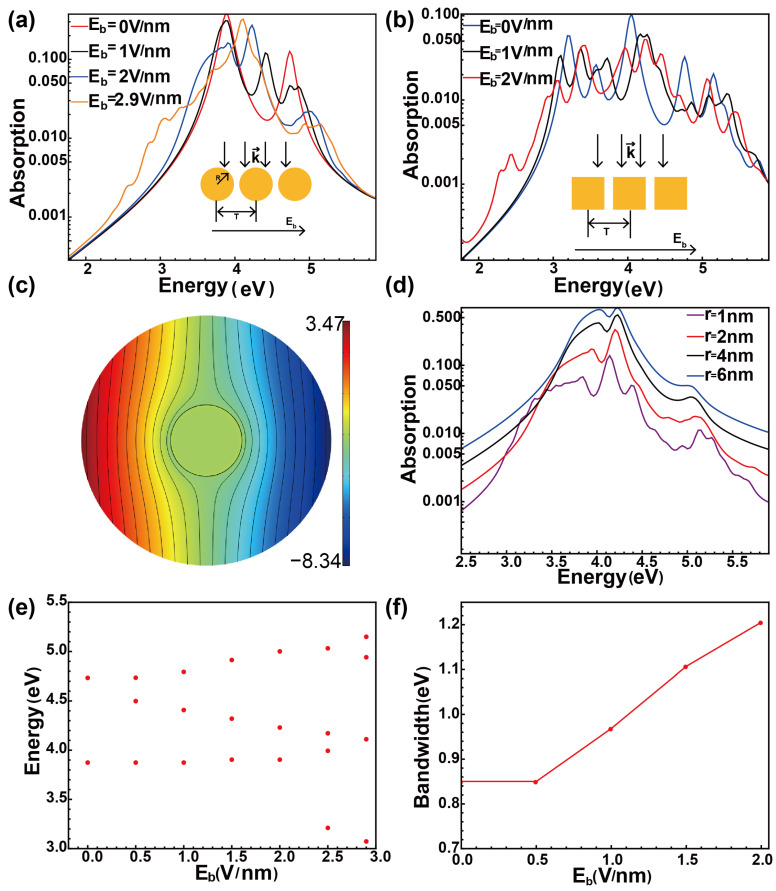
Impact of applying a biased electric field on the optical response of a metasurface. (**a**) Absorption spectra of nanowire array corresponding to different values of biased electric field. The inset represents the schematic of a periodic nanowire array under a DC biased field $E_{b}$. The incident field and biased electric field are in the same direction. *T* is the period of the array, taken as 20 nm. (**b**) Absorption spectra of square nanowire array corresponding to different values of biased electric field. The inset represents the schematic of a periodic square nanowire array under a DC biased field $E_{b}$. (**c**) The potential of the ground state. The solid black lines are the equipotential lines. (**d**) Absorption spectra for different sizes of nanowire array under the same bias. The biased field $E_{b}$ = 2 V/nm. (**e**) Resonance peaks as a function of DC bias. (**f**) Bandwidth of a 2 nm-radius nanowire as a function of the biased field.

A single biased nanoresonator can serve as a foundational building block for constructing a metasurface with tunable bandwidth, as illustrated in the inset in Figure 3a. From the metamaterials’ point of view, the exotic properties of the plasmonic metasurface highly depend on the unit cell, which is a nanowire in our system. In this configuration, a normally incident field interacts with the metasurface, while the DC bias field is applied horizontally to maximize the bandwidth (as shown in Figure 2c). We consider the ground state of each nanowire to be independent of the other, and Figure 3c illustrates the potential of the ground state. It can be found that the perturbation of the DC field exists only to a limited extent, beyond which the field can be considered uniform. Therefore, the single nanoparticle approximation for the ground state calculation, that is, without considering the coupling and interactions between the units under the bias electric field, is valid as long as the period *T* is larger than this range, simplifying the numerical implementation.

Figure 3a presents the absorption spectra for various bias field strengths, showing a broadening process consistent with the behavior observed for a single nanoresonator. The spectrum at a bias field of 2.9 V/nm, provided to the extent of numerical stability, reveals additional peaks emerging at lower frequencies, indicating further mode splitting and bandwidth broadening. Stronger bias fields are expected to yield even greater bandwidth broadening. Additionally, the metasurface bandwidth can be dynamically tuned by altering the angle between the incident field and the DC-biased field.

To validate the broad applicability of our modulation approach, we systematically analyzed the absorption spectra of square nanowire arrays and individual nanowires with varying dimensions. As shown in Figure 3b, the absorption spectrum of a square nanowire array with a 2 nm side length demonstrates substantial broadening when subjected to an increased bias electric field of 2 V/nm. Moreover, Figure 3d presents the absorption spectra for nanowires with different radii. As the radius increases from 1 nm to 2 nm, 4 nm, and ultimately, 6 nm, the corresponding bandwidths—defined as the energy separation between the leftmost and rightmost peaks—decrease from about 1.95 eV to 1.60 eV, then to 1.01 eV and further to 0.98 eV, revealing a distinct downward trend. Figure 3f further highlights that the bandwidth progressively broadens with increasing bias field strength. Although the phenomenon of surface charging is inherently nonclassical, recent experimental studies have demonstrated its significant impact on the scattering and resonance shift of single plasmonic nanoresonators, even at scales up to several hundred nanometers [18]. This supports the relevance of our bandwidth broadening concept for larger resonators as well. Moreover, in Figure 3e, the variation in the resonance peaks can be clearly seen, including broadening and splitting.

Furthermore, a parallel-plate electrode configuration can realize the biased electric field. A DC bias field is established by charging the upper and lower electrodes, and the nanostructure is not connected with both upper and lower electrode but situated midway between the two parallel plates, ensuring that the structures themselves remain globally charge-neutral. However, the current experiments primarily focus on liquid crystals, as they can achieve significant modulation at lower voltages [31,36,37,38]. In contrast, metal systems require higher voltages to achieve similar modulation and remain an area warranting further experimental investigation.

## 3. Conclusions

In conclusion, we have demonstrated how a DC-biased electric field can broaden the optical bandwidth of a nanoresonator. This phenomenon arises from the redistribution of surface charges induced by the applied bias. Specifically, positively charged regions on the metal surface cause a blueshift, while negatively charged regions induce a redshift, leading to spectral splitting and overall bandwidth broadening. Our findings reveal that the Bennett mode is more sensitive to variations in the bias field compared to the conventional SPP mode, which begins to show significant changes only when the bias field exceeds 2 V/nm. The demonstration of the influence exerted by both structural dimensions and biased field intensity on bandwidth broadening underscores the applicability of this methodology to larger-scale structures. Furthermore, the biased nanoresonator shows promise as a fundamental component for constructing metasurfaces with dynamically tunable bandwidths, opening new opportunities for adaptive nanophotonic applications.

## Data Availability

Data is contained within the article.
